# Physician-Assisted Death: What Everyone Needs to Know

**DOI:** 10.5195/jmla.2018.463

**Published:** 2018-07-01

**Authors:** Mary A. Wickline

**Affiliations:** Medical Center Library, University of California, San Diego, La Jolla, CA

There are certain controversial questions that when asked, we answer immediately and are fairly certain of our position. Are you for or against the death penalty? For or against gun control? For or against abortion? For or against physician-assisted dying? For individuals, the details behind the question—the particulars—are often deeply personal and highly complicated. Our answers, meanwhile, reflect a response to a clearly depicted moral case. Two examples—(1) someone helping a person with a terminal and deeply painful disease be released from misery or (2) someone requesting this service for a rich aunt whose will they are in—represent the easily answered straw men. But the more we actually examine the question, the more complicated it becomes. And there are many resources arguing for and against.

*Physician-Assisted Death: What Everyone Needs to Know* opens with two chapters that are focused on defining terminology that we use casually and think we already understand but may not, as the answers have evolved in the past half century. “How should we think about death and dying?” makes it clear that just defining death has recently become difficult. In the mid-1900s, medicine gained the tools of cardiopulmonary resuscitation (CPR) and mechanical ventilators. These tools complicated the definition of “death,” because, formerly, cessation of respiration and a heartbeat were death. Now, people can be revived and maintained even after all brain function has stopped.

Subsequent chapters take the approach of a debate: the ethical case in favor or against; a history of legal physician-assisted death, which concerns itself primarily with physician liability; options for a legal protocol; the case in favor and the case against legalization. The book ends with the outer edges of non-voluntary physician-assisted death and a final chapter on “How Might Legalization Be Achieved,” which addresses the potential roles of legislatures, the courts, prosecutors, and referenda. The book is supplemented by an appendix with the full text of the Oregon Death with Dignity Act (pp. 205–15).

The author declares in the preface that “the aim of this book is to provide readers with the resources…to take up intelligent and informed positions in this important debate” (p. xiii), and while offering a balanced presentation that includes difficult cases, the author seems to lean in favor of physician-assisted death. Medical librarians will find a remarkable, international array of resources on this subject [1, 2] and will want to purchase a range of titles that represent the full spectrum of the debate. It is highly nuanced. Marcia Angell (former editor of the *New England Journal of Medicine* for about ten years) has a chapter on “The Quality of Mercy” in Timothy E. Quill and Margaret P. Battin’s *Physician-Assisted Dying: The Case for Palliative Care and Patient Choice* [3]. Neil Gorsuch published a legal case against assisted-suicide [4]. Not always, but commonly, authors who use the term “suicide” indicate an alignment against and those who use “death” indicate support.

Atul Gawande ends his book *Being Mortal: Medicine and What Matters in the End* by addressing how complicated the issue is:

At root, the debate is about what mistakes we fear most—the mistake of prolonging suffering or the mistake of shortening valued life…Certainly, suffering at the end of life is sometimes unavoidable and unbearable, and helping people end their misery may be necessary. Given the opportunity, I would support laws to provide these kinds of prescriptions to people. About half don’t even use their prescription. They are reassured just to know they have this control if they need it. But we damage entire societies if we let providing this capability divert us from improving the lives of the ill. Assisted living is far harder than assisted death, but its possibilities are far greater, as well. (pp.244–5) [5]

Gawande goes on to describe and advocate for hospice and palliative care and to remind us that when we did typically die at home, we understood the dying process better:

Technological society has forgotten what scholars call the “dying role” and its importance to people as life approaches its end…This role is, observers argue, among life’s most important, for both the dying and those left behind. (p. 249) [5]

It is a complicated subject—think about suffering infants or an Alzheimer’s patient who has previously declared a wish not to live with impaired cognitive ability yet is physically healthy while suffering severe memory loss. It is a horrifyingly painful thing to have to say, “Let my loved one die.”

Much of the discussion focuses on physicians’ and clinicians’ liabilities, concerns, and values, because it is legally dangerous for any person to be with a patient using assisted death. As a consequence, the patient is forced to die alone—something none of us want for ourselves or those we love. Patients are less afraid if they know what can be done and are then able to be with their loved ones.

There are a multitude of resources on this subject, both narrowly and broadly. The National Academies of Sciences, Engineering, and Medicine (NASEM) sponsored a public, open debate in February 2018 that can viewed as a webinar on their website.

*Physician-Assisted Death* is recommended for academic medical centers and hospital libraries. It can be useful to libraries or college courses as a textbook introduction that addresses ethics and pro and con arguments. There are other books that would be good companion titles to this one. Two by physician authors, in particular, have deeper, philosophical introductions to this discussion. Atul Gawande’s *Being Mortal* and Haider Warraich’s *Modern Death: How Medicine Changed the End of Life* [6] address the subject in a context of palliative care. Much of what seems most critical is teaching physicians how to talk about dying.
